# Uncertainty and the unmet informational needs of patients with cancer of unknown primary (CUP): a cross-sectional multi-site study

**DOI:** 10.1007/s00520-022-07228-7

**Published:** 2022-07-09

**Authors:** Lisa Guccione, Krista Fisher, Linda Mileshkin, Richard Tothill, David Bowtell, Stephen Quinn, Anna DeFazio, Chris S. Karapetis, Nicholas Wilcken, Madhu Singh, Christopher Steer, Bo Gao, Mark Warren, Ian M. Collins, Narayan Karanth, Cindy Bryant, Penelope Schofield

**Affiliations:** 1 Department of Health Services Research, Peter MacCallum Cancer Centre, 305 Grattan Street, Melbourne, Victoria 3000 Australia; 2 University of Melbourne, Melbourne, VIC Australia; 3 Swinburne University of Technology, Melbourne, VIC Australia; 4 Westmead Institute for Medical Research, Sydney, NSW Australia; 5 Department of Gynaecological Oncology, Westmead Hospital, Westmead, NSW Australia; 6 University of Sydney, Sydney, NSW Australia; 7 Flinders Medical Centre, Bedford Park, South Australia Australia; 8 Flinders University, Adelaide, South Australia Australia; 9 Barwon Health, Andrew Love Cancer Centre, Geelong, VIC Australia; 10 Border Medical Oncology, Albury Wodonga Regional Cancer Centre, Albury, NSW Australia; 11 Bendigo Health, Regional Melbourne, Victoria, Australia; 12 SouthWest Healthcare, Warrnambool, VIC Australia; 13 Top End Health Service, Darwin, NT Australia

**Keywords:** Cancer, Oncology, Cancer of unknown primary, CUP, Quality of life, Supportive care, Cancer experiences

## Abstract

**Objective:**

This study aimed to determine the healthcare experiences, quality of life, and psychosocial needs of patients with cancer of unknown primary (CUP) early after diagnosis; comparing their experiences to patients with advanced cancer of a known primary (non-CUP control patients) and published general population reference data where available.

**Methods:**

This study was a cross-sectional, multi-site study comparing CUP patients (*n* = 139) compared to non-CUP controls (*n* = 45). Demographic, clinical information and patient-reported outcome questionnaire data were collected at baseline.

**Results:**

Differences in healthcare experienced were found between CUP and non-CUP controls with CUP patients reporting higher scores for unmet medical communication/information needs compared with non-CUP control patients (*p* = 0.013) as well as greater uncertainty in illness (*p* = 0.042). Whilst no differences were found between CUP and non-CUP controls on the EORTC and PROMIS measures, of those that ‘received written information about your cancer…’ and asked ‘…how useful was it?’ fewer CUP patients reported finding the information useful 40% vs 61%, and more were likely to not have received written information at all 59% vs 32%; (*p* = 0.002). Additionally, of those that found information about their cancer online, fewer patients with CUP reported finding it useful 32% vs 48% control patients (*p* = 0.005).

**Conclusions:**

CUP patients have unmet medical communication/information needs and greater uncertainty in illness but do not differ in health-related quality of life domains compared to patients with advanced cancer of a known primary.

## Introduction

Cancer of unknown primary (CUP) is a diagnosis of metastatic disease, whereby following extensive investigation, a primary anatomical site cannot be identified. Although considered rare, CUP accounted for approximately 1.8% of all cancers diagnosed in 2019 and is the 4th most common cause of cancer death worldwide, with only a 13% chance of surviving at least 5 years [1]. The majority of CUP tumours are adenocarcinomas and are most frequently described histopathologically as being poorly differentiated [2] with treatment for these tumours largely restricted to chemotherapy [3]. In comparison to patients with metastatic cancer of a known primary site, CUP patients have significantly lower survival overall [4].

It is clear from the evidence derived from patients of known primary cancers that patients benefit from screening and provision of supportive care interventions [5]. The depression and feelings of hopelessness experienced by those with incurable cancer [6] are only further compounded when the diagnosis is uncertain [7]. Yet in spite of this, and with relatively poor prognosis and few effective treatments available, there is minimal published research to identify and understand the unique psychological and supportive care needs for patients diagnosed with CUP.

A small qualitative study (ten patients) suggested that those diagnosed with CUP struggle mostly with the uncertainty and distress regarding their prognosis [7]. A larger quantitative study (50 CUP patients compared to 162 patients with either metastatic breast or colorectal cancer) suggested that those diagnosed with CUP experience comparatively higher depression and anxiety and poorer quality of life; however, key clinical factors such as time since diagnosis and stage of disease were not controlled for in sampling [8]. Moreover, metastatic breast and colorectal cancers have a considerably long median survival than CUP [9], hence are not optimal comparison groups.

In the only large-scale study to explore experiences of care, it was found that CUP patients in the UK require more psychosocial support and supportive care interventions that will target patient understanding of their diagnosis to help manage the uncertainty and complex trajectories of care that are typical of CUP [10]. However, CUP patients were defined using classification codes generated from administrative data records hence if a site of origin was identified during the illness trajectory, this change might not have been recorded, resulting in some patients with a definitive diagnosis being classified as CUP. It is also likely that the sample was mostly comprised of CUP patients with a favourable subtype of CUP, who have higher survival rates [10]. Given the limitations of these studies, there is a clear need to conduct prospective studies on larger CUP cohorts that are more representative of the population. In addition, to develop and test effective supportive care services for patients with CUP and their families in Australia, we need to understand their experiences and identify the unique psychological and supportive care needs of this patient population.

The aim of this study was to compare the psychological distress, quality of life, unmet needs, symptoms, uncertainty and hopelessness reported by people with CUP, to those with advanced cancer of a known primary (non-CUP patients) diagnosed within 2 months. This study also compared CUP patient quality of life measures with a general population data comparison group.

## Methods

### Study design

This study was a cross-sectional study design comparing the experiences of known primary site cancer patients (non-CUP patients) with unknown primary site cancer patients (CUP patients) within 2 months diagnosis. Non-CUP participants were recruited through a research project, Understanding the experience of advanced cancer for known and unknown primaries between years 2013 and 2018 CUP participants comprised a sub-set of patients enrolled within 2 months of diagnosis, recruited through a parallel research project, SUPER: Solving Unknown Primary CancER between years 2013 and 2018. Ethics approval was obtained from the Human Research Ethics Committee (HREC) for both projects. De-identified data is available upon request.

### Patient involvement

CUP consumer representatives were collaboratively involved in the design and execution of the study via involvement in the application of funding and the interpretation and write-up of the results. Consumer representatives are those that have an interest in the results of the study but are not researchers themselves and can provide valuable insight into the prioritisation and research agenda-setting, contribute to study design, outcomes and material, and especially valuable in prioritising the selecting outcome measures that matter to patients and providing feedback on appropriate language. CUP consumers were involved in the application of funding and the interpretation and write-up of the results.

### Participants

#### CUP participants

The CUP participants comprised a sub-set of participants recruited through the Solving Unknown Primary CancER (SUPER) project. This sub-set was limited to SUPER patients diagnosed within 0 to 2 months of study enrolment. The SUPER project is a prospective longitudinal cohort study of *n* = 296 CUP patients that collected clinical, psychosocial data, and participant tumour samples for molecular analyses. Patients were eligible for the SUPER study if they (i) were diagnosed with cancer of no confirmed primary site, having undergone preliminary diagnostic work-up, (ii) had not commenced treatment more than 6 months ago and (iii) were able to read and write in English and provide informed consent. Patients under the age of 18 years old, an ECOG performance status greater than or equal to three or with uncontrolled medical or psychological conditions were excluded. The CUP participant group for the current study were considered eligible for inclusion if they (i) had been diagnosed with advanced cancer with an unknown primary site within 0–2 months from study enrolment and (ii) had complete patient-reported outcome measures (PROM) and clinical data at the baseline.

#### Non-CUP participants

The non-CUP participants were outpatients with advanced cancer (stage 4 metastatic disease), across a range of different tumour streams including lung, colorectal, gastro-intestinal, gynaecological and head and neck cancers. These tumour sites were selected as the sites from which evidence suggests CUP most commonly arises [11]. Eligible non-CUP participants had (i) a diagnosis of advanced cancer with a known primary tumour site, (ii) metastatic disease diagnosed within 0 to 2 months of study enrolment and (iii) ability to read and write in English and provide informed consent.

### Recruitment

For both CUP and non-CUP participants, research staff pre-screened and identified potentially eligible patients across different tumour streams. Once patients were identified, the researcher confirmed their eligibility with their treating clinician. If deemed eligible, the patient was approached to introduce and determine their interest in the study. Interested patients were given a Participant Information Sheet and Consent Form (PICF) and an opportunity to ask and clarify any questions they may have. Written consent was then requested from the patient, and once consent was obtained, they were given a copy of the PICF, baseline questionnaire and a reply-paid envelope.

### Measures

#### Demographic and clinical information

Clinical and demographic data were collected by an experienced researcher via medical record audits. Demographic information included participant gender, age, marital status, current employment, level of education and nationality. Clinical data collected for the non-CUP patients included date of diagnosis, date of metastatic disease, primary and metastatic tumour sites, primary treatment details, ECOG performance status (ECOG 1: symptomatic but completely ambulatory; ECOG 2: ambulatory and capable of self-care, < 50% of waking hours in bed; ECOG 3:only limited self-care, > 50% of waking hours confined to bed or chair; ECOG 4: completely disabled and confined to bed or chair; ECOG 5: deceased; [12]) and past history of cancer. Comparative clinical data was obtained for the CUP cohort from the SUPER dataset with the addition of suspected primary site.

#### Patient reported outcome questionnaire

Patient-reported outcome data was collected within 1 month of study enrolment. Questionnaires were reviewed for completeness of data, and missing data were followed up with the participants.

#### Cancer-specific health-related quality of life

The EORTC QLQ-C30 is a 30-item self-report measure incorporating five functional scales (physical, role, cognitive, emotional and social functioning), three symptom scales (fatigue, pain, nausea/vomiting), a global health status (GHS) scale and six single items assessing dyspnoea, sleep disturbance, appetite loss, constipation, diarrhoea and financial impact [13]. Its reliability and concurrent and criterion validity have been demonstrated in numerous studies [13–15], and it is more acceptable to CUP patients than alternatives [16]. Cronbach’s alpha coefficient values range from 0.72 to 0.95, illustrating the reliability of the scales measured. Interscale correlations were statistically significant (*p* < 0.05), indicating clinical validity of the data collected [17].

#### Psychological morbidity, symptoms and functioning

The Patient-Reported Outcomes Measurement Information System (PROMIS®) short forms were used to collect data on the following health-related quality of life domains: anxiety, depression, fatigue, pain interference, pain intensity, sleep disturbance, physical function, satisfaction with social roles and activities. All relevant short forms were specifically developed for use in clinical oncology research and are standardised, accurate and efficient self-report measures [18]. High Cronbach’s alpha values (0.86–0.96) were measured for each PROMIS scale and were sufficiently unidimensional [19].

#### Uncertainty in illness

The 23-item Mishel Uncertainty in Illness Scale-Community Form (MUIS-C) represents an abbreviated version of the 32-item Mishel Uncertainty in Illness-Adult (MUIS-A) form [20]. As the original MUIS-A scale was developed for hospitalised or acutely ill adults, the MUIS-C scale was developed for use in chronically ill persons who are not likely to be hospitalised and may not be receiving medical treatment [20]. The MUIS-C has been used with patients diagnosed with a variety of chronic conditions including cancer [21–24]. Patients use a 5-point Likert-type scale to respond to items. Responses are summed to create a total score. Higher total scores indicate greater uncertainty. Items comprising the MUIS-C have demonstrated moderate to high internal consistency (alpha = 0.74 to 0.92) across patient groups [20].

#### Hopelessness

The 8-item Hopelessness Assessment in Illness (HAI) questionnaire was developed to measure hopelessness specifically for patients with a terminal illness [25]. Validation analyses of the eight-item scale revealed considerable internal consistency (alpha = 0.87) and convergent validity of the measure.

#### Medical communication/information and psychological needs

The Needs Assessment for Advanced Lung Cancer Patients (NA-ALCP) [26, 27] is a short-form self-reported questionnaire comprising seven domains of needs for patients with advanced incurable cancer. Two domains from this measure were used: Medical communication/information (Med Comm) and psychological/emotional (Psych Emot) needs. These subscales were selected as they cover the two most prevalent needs in advanced cancer populations. They have high internal consistency (alpha = 0.95 and 0.93 respectively) and good divergent and convergent validity [27].

#### Communication about and understanding of illness and treatment (NHS-CPES)

A total of 23 single-items related to patients’ understanding of their diagnosis and treatment, adequacy of communication and experiences with hospital staff were drawn from the UK Department of Health Cancer Patient Experience Survey 2011/2012 [28]. Responses were recoded according to previous research [10].

### Statistical methods

All analyses were conducted using Stata 17 (StataCorp, College Station, Texas). Differences in continuous baseline demographic and clinical data for CUP and non-CUP patients were assessed using *t* tests or Mann–Whitney *U* tests for normally or non-normally distributed data, respectively. Baseline demographic and clinical differences between groups and items in CPES were compared using Pearson’s *χ*^2^ or Fisher’s exact test. Responses between groups to PROM, EORTC, MUIS, HAI, Med Comm and Psych Emot outcomes were assessed using *t* tests. Significant differences between groups were explored using multivariable linear regression, listed in the Results section. Evidence-based guidelines were used to interpret the sizes of between group differences where possible [29]. Results are reported with 95% confidence intervals (95% CI) and a *p* value less than 0.05 (two-tailed) is deemed to be statistically significant. No adjustment has been made for multiple comparisons as this is an exploratory study.

## Results

### Study profile

#### CUP participants

**Fig. 1:**
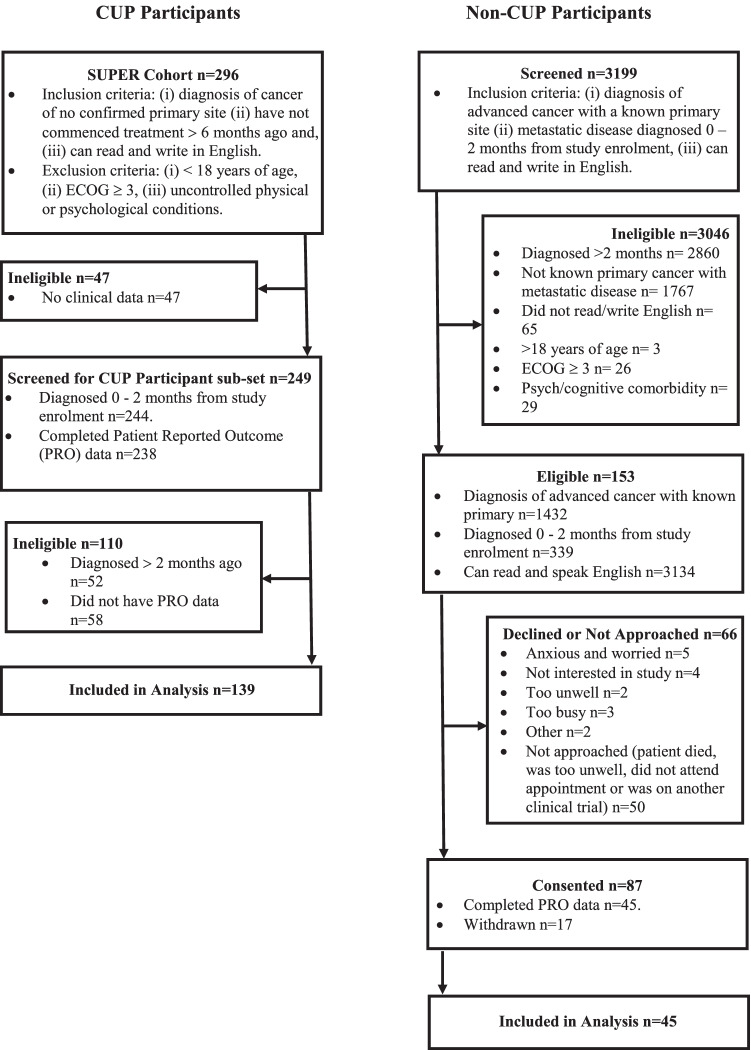
Participant flow diagram

Of the 296 patients recruited to SUPER (consent rate = 92%), 139 with complete clinical and patient-reported outcome data were diagnosed within 2 months of study enrolment and comprise the CUP sample for this study (Fig. 1). The mean time from CUP diagnosis to study consent was 27.1 days (SD = 16.1). From the date of diagnosis to study consent, 32% of CUP participants had received (or were currently receiving) interventional therapy (surgery), 17% radiotherapy and 52% systematic therapy (chemotherapy and/or immunotherapy).

#### Non-CUP participants

Of the 87 patients with metastatic cancer of a known primary site consented to participate in the study (consent rate = 57%), 45 completed clinical and patient-reported outcome data (Fig. 1). The mean time from metastatic diagnosis to study consent was 38 days (SD = 13.1). From the date of diagnosis to study consent, 13% of non-CUP participants had surgery, 29% radiotherapy and 71% systematic therapy (chemotherapy and immunotherapy).

Demographic and clinical information is summarised in Table 1. The mean age of CUP and non-CUP control patients was 60.2 and 62.2 years respectively, with 43% of CUP and 46% of control patients being male. Compared to controls, those with CUP were more likely to have had surgery, 31% vs 13% (*p* = 0.016), and less often received systemic therapy, 51% vs 71% (*p* = 0.019 Table 1). Otherwise, there were no differences in the demographic or clinical variables measured between groups.

**Table 1:** Demographic variables for participants diagnosed with CUP and non-CUP control participants diagnosed with advanced cancer

Variable	CUP *n* (%) or mean (sd) (*n* = 139)	Advanced cancer *n* (%) or mean (*n* = 45)	*p* value
Gender, male	61 (43.9)	21 (46.7)	0.74
Age in years, mean (sd)	60.2 (13.0)	62.2 (11.7)	0.36
ECOG status 0 1 2	43 (31.9) 76 (56.3) 16 (11.9)	10 (22.2) 28 (62.2) 7 (15.6)	0.44
Time from cancer diagnosis to consent in days, mean (sd)	27.1 (16.1)	-	
Suspected site	101 (72.7)		
Has a history of other cancers	38 (27.3)		
Primary site Lung cancers Gynaecological cancers Other cancers Time from primary diagnosis to consent in days, mean (sd)	-	189 (365.9) 7 (16) 11 (24) 27 (60)	
Time from metastatic diagnosis to consent in day, mean (sd)		38 (13.1)	
Current employment situation (s1q2) Working full or part time On sick leave Not employed Retired Home duties Studying Other Missing	21 (15.7) 23 (17.2) 12 (9.0) 63 (47.0) 9 (6.7) 1 (0.7) 5 (3.7) 5 (3.7)	9 (20.9) 8 (18.6) 4 (9.3) 18 (41.9) 2 (4.7) 0 (0.0) 2 (4.7) 0 (0.0)	0.96
Higher level of formal education (s1q4) No formal schooling, primary, secondary Tertiary Trade, TAFE, College	68 (49.3) 42 (30.4) 28 (20.3)	13 (29.5) 17 (38.6) 14 (31.8)	0.056
Non-aboriginal or Torres Strait Islander status	130 (100.0)	45 (100.0)	
English as first language	118 (86.1)	40 (88.9)	0.70
Country of birth Australia Overseas	105 (75.5) 34 (24.5)	28 (62.2) 17 (37.8)	0.083
Metastasis to bone	34 (24.5)	14 (31.1)	0.38
Metastasis to organs	76 (54.7)	31 (68.9)	0.093
Other metastases	38 (27.3)	14 (13.1)	0.77
Interventional treatment received*	44 (31.7)	6 (13.3)	0.016
Radiotherapy treatment received	23 (16.5)	13 (28.9)	0.070
Systemic therapy received*	71 (51.1)	32 (71.1)	0.019

^*^Interventional treatments consisted of surgery. Systemic therapy comprised on chemotherapy, immunotherapy, etc.

### Patient-reported experiences

Analysis of the CPES patient-reported experiences (Table 2) showed a higher percentage of patients with CUP vs controls felt that to some extent, diagnostic tests results were explained in a way that they could understand (98% vs 91%, *p* = 0.018). Of those that ‘received written information about your cancer…’ and asked ‘…how useful was it?’ fewer CUP reported finding the information useful 40% vs 61%, and more were likely to not have received written information at all 59% vs 32%; (*p* = 0.002). Additionally, of those that found information about their cancer online, fewer patients with CUP reported finding it useful 32% compared with 48% control patients (*p* = 0.005). No other significant differences were detected.

**Table 2:** Descriptive statistical comparison of differences between responses for CUP and non-CUP control groups

Variable	Cup *n* (%) (*n* = 139)	Advanced cancer *n* (%) (*n* = 45)	*p* value
**CPES score by item**			
Q1Did a staff member explain the purpose of the test? Yes, to some extent No, but I would have liked an explanation No, but I did not need an explanation	128 (95.5) 1 (0.7) 5 (3.7)	43 (95.6) 2 (4.4) 0 (0.0)	0.075
Q2Did you understand the reasons for the tests you had? Well or very well Not well or not at all	134 (98.5) 4 (2.9)	42 (90.9) 2 (4.5)	0.63
Q3Were the results explained in a way you could understand? Yes, to some extent No, but I would have liked an explanation No, but I did not need an explanation	130 (98.5) 2 (1.5) 0 (0.0)	40 (90.9) 2 (4.5) 2 (4.5)	0.018
Q4Do you feel that you missed out on test that you should have had? Yes No	119 (88.1) 16 (11.9)	40 (93.0) 3 (7.0)	0.57
Q5How do you feel about the way you were told that you had advanced cancer? It was done sensitively It should have been done with more sensitivity It should have been done with a lot more sensitivity	105 (77.2) 24 (17.6) 7 (5.1)	34 (75.6) 7 (15.6) 4 (8.9)	0.66
Q6Did you understand the explanation of what was wrong with you? Yes, I completely understood it Yes, I understood some of it No, I didn’t understand it	73 (53.5) 57 (41.6) 7 (5.1)	24 (53.3) 20 (44.4) 1 (2.2)	0.66
Q7When told about our cancer, were you given written information about the type of cancer you had? Yes, it was easy to understand Yes, but it was difficult to understand No, I was not given written information I did not need written information	29 (21.6) 11 (8.2) 86 (64.2) 8 (6.0)	16 (35.6) 6 (13.3) 21 (46.7) 2 (4.4)	0.13
Q8If you received written information about your cancer, how useful was it? Very or somewhat useful Not very useful or not useful at all I didn’t receive the written information	48 (40.3) 1 (0.8) 70 (58.8)	23 (60.5) 3 (7.9) 12 (31.6)	0.002
Q9Overall, how well do you feel you understand your cancer? Well or very well No so well or not well at all	92 (67.2) 45 (32.8)	37 (82.2) 8 (17.8)	0.06
Q10Overall how well do you feel your doctor understands your cancer? Well or very well No so well or not well at all	106 (80.9) 25 (19.1)	40 (93.0) 3 (7.0)	0.092
Q11Have you had discussions about the possible treatment options with your doctor in the last 3 months? Yes No	22 (16.2) 114 (83.8)	7 (15.6) 38 (84.4)	1.0
Q12Were you given the right amount of (written or verbal) information about your treatment options? Too much information The right amount of information Not enough information	4 (3.5) 98 (85.2) 13 (11.3)	0 (0.0) 35 (62.1) 3 (7.9)	0.44
Q13Have you been involved as much as you wanted to be in decisions about your care and treatment? Yes, definitely Yes, to some extent No, but I would like to be more involved	81 (60.9) 44 (33.1) 8 (6.0)	30 (69.8) 10 (23.3) 3 (7.0)	0.44
Q14Have you been provided with the name of a Specialist nurse/care co-ordinator who would be in charge of your care? Yes No	62 (45.6) 74 (54.4)	12 (29.3) 29 (70.7)	0.072
Q15When you have important questions to ask your specialist nurse/care coordinator, how often do you get answers that you can understand? All or most of the time Some of the time Rarely or never I do not ask any questions	54 (73.0) 6 (8.0) 2 (3.0) 12 (16.0)	19 (70.4) 3 (11.1) 1 (3.7) 4 (14.8)	0.94
Q16Have hospital staff given you information about support or self-help groups for people with cancer? Yes No, but I would have liked this information It was not necessary	62 (48.4) 36 (28.1) 30 (23.4)	27 (62.8) 9 (20.9) 7 (16.3)	0.29
Q17When you have important questions to ask your cancer doctor, how often do you get answers that you can understand? All or most of the time Some of the time Rarely or never I do not ask any questions	114 (85.1) 16 (11.9) 2 (1.5) 2 (1.5)	37 (84.1) 5 (11.40 1 (2.3) 1 (2.3)	1.0
Q18Do you ever think that your cancer doctor or nurse is deliberately not telling you certain things that you want to know? Often Sometimes Only once Never	4 (3.0) 18 (13.3) 5 (3.7) 108 (80.0)	0 (0.0) 7 (15.9) 1 (2.3) 36 (81.8)	0.82
Q19Are you able to discuss any worries or fears with hospital staff? As much as I wanted or most or some of the time Not at all, but I would have liked to I do not have any fears	119 (90.8) 2 (1.5) 10 (7.6)	43 (97.7) 0 (0.0) 1 (2.3)	0.40
Q20If your family or someone else close to you wants to talk to your cancer doctor, do they have enough opportunity to do so? Yes, definitely or to some extent No No, my family or friends are involved or do not want to talk to the doctor or I do not want them to talk to the doctor	130 (94.2) 5 (3.6) 3 (2.2)	39 (88.6) 1 (2.3) 4 (9.1)	0.13
Q21Has your cancer doctor or nurse specialist given your family or someone close to you all the information they need to help care for you at home? Yes, definitely or to some extent No No, my family or friends are involved or do not want to talk to the doctor or I do not want them to talk to the doctor	100 (74.1) 28 (20.7) 7 (5.2)	30 (68.2) 11 (25.0) 3 (6.8)	0.70
Q22Have you or your family tried to access information about your cancer online? Yes, and it was easy to understand Yes but it was difficult to understand or I/we did not want the information No, I/we did not try to access information	42 (31.8) 35 (26.5) 55 (41.7)	20 (47.6) 8 (19.0) 14 (33.3)	0.17
Q23If you found information about your cancer online, how useful was it? Very or somewhat useful Not very useful of not useful at all Didn’t get any information	52 (43.0) 17 (14.0) 52 (43.0)	29 (70.7) 1 (2.4) 11 (26.8)	0.005

Note: Some options in items have been merged and others been deleted

### Patient-reported outcomes

Descriptive and group comparison statistics for EORTC scales, PROMIS scales, MUIS-C, HAI and MedComm and Psych Emot scales are provided in Table 3. Comparisons between the CUP patients and general population reference values are also provided in Table 3.

**Table 3:** Descriptive statistical comparison (all results are from *t* tests) of differences between responses for CUP and non-CUP control groups on the PROMIS, EORTC, MUIS-C, HAI, Med Comm and Psych Emot needs measures. Descriptive statistical comparison of the CUP sample with general population reference data on the EORTC

PROMIS outcome	Cup (*n* = 139)	Advanced cancer (*n* = 45)	*β* (95% CI)	*p* value	Effect (Cohen’s d)
Anxiety	57.5 (10.1)	55.8 (10.1)	1.70 (− 1.72, 5.12)	0.33	0.17
Depression	53.2 (8.9)	52.3 (10.1)	0.88 (− 2.24, 4.01)	0.57	0.10
Fatigue	56.4 (8.6)	54.0 (8.8)	2.40 (− 0.53, 5.32)	0.11	0.28
Pain interference	55.9 (10.3)	53.8 (9.5)	1.73 (− 1.28, 5.55)	0.22	0.21
Pain intensity	45.2 (10.0)	41.8 (9.5)	3.41 (− 0.04, 6.85)	0.052	0.34
Sleep disturbance	53.1 (9.4)	52.2 (9.4)	0.94 (− 2.28, 4.16)	0.56	0.10
Physical function	41.6 (8.7)	42.9 (8.2)	− 1.28 (− 4.21, 1.65)	0.39	0.15
Satisfaction with social roles and activities	44.8 (11.1)	47.0 (10.0)	− 2.16 (− 5.87, 1.53)	0.25	0.20
EORTC outcomes					
Global health status	57.7 (24.0)	64.7 (23.1)	− 7.47 (− 15.66, 0.72)	0.073	0.31
Physical functioning	70.7 (25.2)	75.1 (22.6)	− 4.42 (− 12.99, 4.16)	0.31	0.18
Role functioning	56.0 (34.1)	54.8 (32.4)	1.27 (− 10.44, 13.00)	0.83	0.04
Emotional functioning	71.5 (22.4)	73.3 (25.6)	− 1.78 (− 9.84, 6.28)	0.66	0.08
Cognitive functioning	76.3 (20.8)	77.1 (24.1)	0.87 (− 8.33, 6.58)	0.82	0.04
Social functioning	63.1 (30.1)	61.2 (29.9)	1.83 (− 8.53, 12.19)	0.73	0.06
Fatigue	47.8 (24.4)	42.3 (29.4)	5.46 (− 3.46, 14.38)	0.23	0.21
Nausea/vomiting	17.5 (23.3)	10.3 (15.2)	7.19 (− 0.34, 14.73)	0.061	0.33
Pain	35.3 (32.8)	30.2 (25.0)	5.11 (− 5.75, 15.96)	0.35	0.16
Dyspnoea	27.3 (29.5)	29.4 (33.1)	− 2.11 (− 12.68, 8.45)	0.69	0.07
Insomnia	41.9 (31.9)	35.7 (28.9)	6.19 (− 4.69, 17.09)	0.26	0.20
Appetite loss	37.2 (34.1)	28.6 (30.9)	8.65 (− 2.96, 20.27)	0.14	0.26
Constipation	30.0 (34.0)	24.6 (29.5)	5.34 (− 6.14, 16.83)	0.36	0.16
Diarrhoea	14.2 (25.2)	8.7 (23.4)	5.49 (− 3.15, 14.12)	0.21	0.22
Financial problems	21.3 (29.2)	30.2 (35.3)	− 8.89 (− 19.49, 1.71)	0.10	0.29
MUIS-C total	64.0 (13.9)	58.9 (13.5)	5.13 (0.19, 10.08)	0.042	0.37
HAI total	3.9 (3.1)	4.3 (3.4)	− 0.46 (− 1.53, 0.60)	0.39	0.15
Med Comm total	2.8 (1.0)	2.3 (1.1)	0.45 (0.10, 0.80)	0.013	0.44
Psych Emot total	2.6 (0.9)	2.3 (0.9)	0.26 (− 0.05, 0.57)	0.10	0.29
EORTC outcomes	General population	Cup (*n* = 139)	*β* (95% CI)	*p* value	Effect (Cohen’s d)
Global health status	66.1 (21.7)	57.3 (24.0)	(53.2, 61.3)	< 0.001	0.37
Physical functioning	85.1 (18.9)	70.7 (25.2)	(66.4, 74.9)	< 0.001	0.57
Role functioning	84.3 (24.6)	56.0 (34.1)	(50.3, 61.8)	< 0.001	0.83
Emotional functioning	74.2 (24.7)	71.5 (22.4)	(67.7, 75.3)	0.16	0.12
Cognitive functioning	84.8 (21.3)	76.3 (20.8)	(72.8, 79.7)	< 0.001	0.41
Social functioning	86.2 (24.1)	63.1 (30.1)	(58.0, 68.1)	< 0.001	0.77
Fatigue	29.5 (25.5)	47.8 (24.1)	(43.7, 51.9)	< 0.001	0.75
Nausea/vomiting	5.9 (1.0)	17.5 (23.3)	(13.6, 21.4)	< 0.001	0.50
Pain	23.5 (27.1)	35.3 (32.8)	(29.7, 40.8)	< 0.001	0.36
Dyspnoea	15.9 (24.6)	27.3 (29.5)	(22.3, 32.2)	< 0.001	0.38
Insomnia	26.6 (30.3)	41.9 (31.9)	(36.5, 47.3)	< 0.001	0.48
Appetite loss	10.0 (21.6)	37.2 (34.1)	(31.5, 43.0)	< 0.001	0.80
Constipation	12.5 (23.3)	30.0 (34.0)	(24.2, 35.7)	< 0.001	0.51
Diarrhoea	9.5 (20.9)	14.2 (25.2)	(9.9, 18.5)	0.031	0.19
Financial problems	10.6 (23.6)	21.3 (29.2)	(16.4, 26.2)	< 0.001	0.37

Note: The beta coefficients represent the effect of group assignment on each outcome using a *t* test

There were no statistically significant differences between CUP and non-CUP control participants in any PROMIS scales or the EORTC QLQ C30 scales. Differences were found in the scores of unmet medical communication/information needs scale between CUP patients and non-CUP controls but not the unmet psychological needs scale (Table 3). CUP patients reported higher scores for unmet medical communication/information needs compared with non-CUP control patients (*p* = 0.013). CUP patients, compared to non-CUP control patients, also reported greater uncertainty in illness (*p* = 0.042).

When compared to the general population reference values [30], CUP patients differed significantly in almost every EORTC subscale as expected. All functioning outcomes were less than of the general population (*p* < 0.001), apart from emotional functioning (*p* = 0.16). They experienced significantly greater levels of all clinical outcomes from fatigue to diarrhoea (*p* < 0.001 apart from diarrhoea with *p* = 0.031). CUP patients also experienced greater financial problems (*p* < 0.001) than the general population.

## Discussion

The current study aimed to determine the patient experiences, quality of life and psychosocial needs that are unique to patients at early diagnosis with CUP. No differences in demographic and clinical variables were found between CUP and non-CUP control patients at within 2 months of diagnosis; however, these groups did differ in the proportions of patients receiving different types of treatment. There was a significant difference in the percentage of patients receiving interventional treatments, with more CUP patients receiving surgery compared to non-CUP control patients (31% vs 13%). In contrast, more non-CUP control patients were undergoing systemic therapies than CUP patients (71% vs 51). Whilst these differences are important, it is essential to note that these were recorded within2 months of diagnosis and are therefore more likely to be attributed to the complex nature of diagnosing and treating patients with CUP compared to the treatment pathways when the primary site of cancer is known.

Results predominantly indicated that patients with a diagnosis of CUP experience significantly greater informational needs and uncertainty in illness compared to those patients with cancer of a known primary. The literature suggests that the need for information for people with cancer is greatest after diagnosis and at the start of treatment, decreasing over time after that [31]. Yet, more than half of the patients with CUP reported not receiving any written information about their cancer. Furthermore, when patients did receive written information, only 40% reported that this information was useful; similarly, the case with information available online, 43% of patients with CUP found this information useful compared to 70% of patients seeking information on cancer of a known primary. These findings reflect the experiences of patients with rare cancers [32]. Like those with rare cancers, patients with CUP appear to be overlooked in the provision of adequate information resources and supportive care interventions. Although written and on-line resources for CUP patients are available in Australia, the resources are scant compared with other types of cancer, and the content of these resources does not appear to be adequately meeting the needs of patients or clinicians.

The importance of information when diagnosed with cancer is well established in the literature [33, 34]. Reportedly, patients who are poorly informed about their cancer are less likely to participate in medical decision making and are also more likely to experience greater uncertainty and anxiety as well as seek alternative therapies that lack scientific evidence [35, 36]. CUP is a cancer diagnosis which few in the community have heard of [37], has limited treatment options [2, 3, 38, 39] and dismal survival outcomes [40]. Lack of information for patients with CUP serves to further compound difficulties faced by patients. As observed amongst more common cancer types, utilisation of informative online information resources and supportive services have been found to show increased hope, positive emotions [41] and improved psychological well-being [42].

Associations have been found between information needs, the usefulness of information and increasing uncertainty [43–45]. Uncertainty is a well-documented experience and a common feature of people with cancer [43]. Patients who experience uncertainty in illness identify factors that contribute to their uncertainty as either ambiguity regarding their state of illness, perceived complexity about treatment and their system of care, unpredictability of the course and outcome of their illness or inadequate information about their illness [46]. Previous research exploring predictors of uncertainty in cancer patients specifically have found an association between increasing uncertainty and information needs for patients who have undergone surgery for colon cancer [44]. This study found that those patients who placed the greatest emphasis on information to help them manage post-surgery also reported the greatest uncertainty in their illness [44]. Furthermore, the perceived ‘quality of information’ has also been found to influence uncertainty in illness. Patients with breast cancer that are most satisfied with the quality of information they have received also report experiencing less uncertainty in comparison to those that are least satisfied [45].

Whilst patients with a CUP diagnosis did not differ from non-CUP control patients in quality of life, hopelessness, emotional distress, pain intensity, sleep disturbance, physical function, satisfaction with social roles and activities and psychological/emotional needs. Compared to general population reference data [30], significant differences across all subscales except for emotional functioning were reported. Medium to large-sized differences were found in the physical, emotional, cognitive and social functioning subscales as well as appetite loss and constipation, which may suggest areas of greatest burden. Only minimal differences were observed with mild to moderate anxiety, fatigue and pain interference being reported by the CUP sample relative to normative standards [47].

Over half of CUP patients reported not receiving any written information about their disease. The usefulness of either written information received or online information sought by CUP patients or their families was significantly less than that being reported for patients who were diagnosed with cancer of a known primary. The lack of adequate information for patients with CUP may also be attributed to significantly higher uncertainty in illness compared to non-CUP control patients. Given that uncertainty is considered to be a major stressor that people seek to reduce [48], interventions that aim to minimise uncertainty in CUP patients should target optimizing the provision of high quality information tailored to CUP patients’ individual needs, especially for those that have the greatest information needs.

### Limitations

Initial attempts were made to frequency match the non-CUP control patient sample to a sub section of the complete SUPER CUP patient sample based on criteria of dominant metastatic site, place of residence (rural/regional) and treatment intent; with eligibility being that participants have received a diagnosis between 0 and 2 months (initial diagnosis for CUP patients and metastatic diagnosis for controls). However, due to the very large number of patients that were required to be screened to assess eligibility, a decision was made to abandon frequency matching which would have resulted in a comparison of CUP *n* = 45 and control *n* = 45, and instead to use the complete larger SUPER CUP patient sample for a more robust analysis, (CUP *n* = 139). In addition, both samples were limited to people who were proficient in English. Studies of people with cancer from culturally and linguistically diverse backgrounds (CALD) indicate that they experience poorer quality of life, and greater psychological morbidity [49]. It is expected that this relationship may be greater amongst those from CALD backgrounds diagnosed with CUP.

### Clinical implications

These results indicate that there is a notable paucity of information resources that meet the needs of patients with CUP compared to advanced cancer patients with a known primary site at early diagnosis. Furthermore, the lack of information available when desire for information is likely to be greatest early after diagnosis may in turn also contribute to the greater uncertainty experience by patients with CUP. These findings may inform future resources for patients with CUP and the implementation of new clinical care guidelines that include the dissemination of patient resources to this group especially post diagnosis. We have also identified that uncertainty is the greatest psychological burden for patients with CUP. Considered a major psychological stressor for patients with cancer [48], the modulation of uncertainty through the provision of information resources tailored to the unique needs of individuals diagnosed with CUP. As a result of these findings, we are co-designing an interactive educational website with clinicians, patients and carers to be made available to CUP clinicians, carers and patients who have metastatic disease but the primary site cannot be identified. Using the latest technical developments, this web-based platform will deliver information, education and resources and support available in an easily digestible and tailored form for clinicians, patients and families incorporating written, multimedia and graphical formats, at a location and a time of their convenience.

## Conclusions

Taken collectively, these findings allow important conclusions to be drawn for the future direction of improving the care experiences and outcomes for patients with CUP. These findings will enable the design and testing of a psycho-educational, supportive care intervention package that can aim to reduce the great uncertainty associated with CUP by the provision of information and support resources that are specific to CUP.

## Data Availability

De-identified data is available upon request.
